# Advances in fear memory erasure and its neural mechanisms

**DOI:** 10.3389/fneur.2024.1481450

**Published:** 2025-01-06

**Authors:** Wenbo Guo, Xibo Wang, Zihan Zhou, Yuhui Li, Yani Hou, Keyan Wang, Ruyuan Wei, Xiaoyu Ma, Hao Zhang

**Affiliations:** ^1^Institution of Traditional Chinese Medicine Innovation Research, Shandong University of Traditional Chinese Medicine, Jinan, China; ^2^Department of Second Clinical Medical School, Cheeloo College of Medicine, Shandong University, Jinan, China; ^3^Department of Public Health School, Cheeloo College of Medicine, Shandong University, Jinan, China; ^4^Department of Rehabilitation, Medical School, Shandong Yingcai University, Jinan, China; ^5^School of Health Sciences, Shandong University of Traditional Chinese Medicine, Jinan, China; ^6^School of Anesthesia, Xuzhou Medical University, Xuzhou, China; ^7^Department of Neurology, The Second Hospital of Shandong University, Jinan, China

**Keywords:** fear memory, memory erasure, neuroplasticity, engram cell, perineuronal net

## Abstract

**Background:**

In nature, animals must learn to recognize danger signals and respond immediately to threats to improve their environmental adaptation. However, excessive fear responses can lead to diseases such as post-traumatic stress disorder, wherein traumatic events result in persistent traumatic memories. Therefore, erasing pathological fear memories *in vivo* is a crucial topic in neuroscience for understanding the nature of memories and treating clinically relevant diseases.

**Main text:**

This article reviews recent studies on fear memory erasure, erasure of short- and long-term memory, fear memory erasure and neuroplasticity, the neural circuitry and molecular mechanisms of fear memory erasure, and the roles of engram cells and perineuronal nets in memory erasure.

**Conclusion:**

Research on the mechanism of memory erasure is limited, and a plausible explanation for the essential difference between memory erasure and memory extinction still needs to be provided. Notably, this review may guide future studies on fear memory and its underlying molecular mechanisms, which may help to develop novel treatment strategies for post-traumatic stress disorder, anxiety, and other mental disorders.

## 1 Introduction

The factors that cause a fear response to develop into a pathological fear memory after a person experiences the same trauma are poorly understood. However, erasing fear memories in patients is important once they have been formed (1, 2). Notably, understanding fear memory erasure and the treatment of disorders caused by fear memories requires elucidating three questions: (i) Can fear memories be erased? (ii) What is the best way to erase fear memories? and (iii) What are the mechanisms of fear memory erasure?

## 2 Fear memory erasure

The erasure of pathological fear memories has mainly relied on exposure therapy, which involves exposing the patient to fear-inducing stimuli in a completely safe environment to diminish their fear response to traumatic stimuli. However, the actual effectiveness of exposure therapy in clinical practice is less than optimal, and patients undergoing this therapy experience the relapse of fear memories frequently. Animal studies on exposure therapy typically employ memory extinction, where animals are repeatedly presented with a conditioned stimulus (CS) without the unconditioned stimulus (US), leading the animal to “forget” the existing conditioned reflex. Nonetheless, studies in rats and mice undergoing extinction training for conditioned fear memories have shown that these memories can resurface under certain conditions. Notably, the memories can reappear and may be challenging to erase when a period has elapsed after the training, when the animals are re-exposed to fear-inducing stimuli, or when they return to the “unsafe environment” where the fear memory was formed, indicating that simple extinction training may not entirely erase fear memories in the brain. These processes are known as spontaneous recovery, reinstatement, and renewal of fear memories, respectively (3, 4). Subsequently, this results in the formation of a new “extinction memory” that competes with and suppresses the original memory trace. Therefore, spontaneous recovery, reinstatement, and renewal serve as indicators to assess whether memory has been erased. Consequently, this also explains why patients with post-traumatic stress disorder (PTSD) treated with exposure therapy often experience relapse, making subsequent treatment more difficult. Therefore, research has focused on erasure, instead of only extinction, of fear memories.

### 2.1 Methods of fear memory extinction

Although studies on young mice have reported that simple memory extinction can erase fear memories (5), studies on adult mice have shown that simple extinction training is ineffective in erasing fear memories. Notably, two fundamental stages have been identified during memory formation: (i) consolidation, where the memory transitions from short- to long-term memory upon initial formation, and (ii) reconsolidation, where a previously consolidated memory becomes labile after retrieval and needs to be reconsolidated (6, 7). Memories are labile and susceptible to interference during these two stages. Based on this theory, two main behavioral methods—immediate and post-retrieval extinctions—have been proposed for erasing fear memories, corresponding to the time windows for memory consolidation and reconsolidation (8–10).

#### 2.1.1 Immediate extinction

Based on the memory consolidation theory, Myers et al. were the first to explore whether extinction performed during consolidation after memory acquisition could effectively erase fear memories. They subjected rats to fear-potentiated startle conditioning and conducted memory extinction training at various time points after memory acquisition (10 min, 1 h, and 72 h). Subsequently, they tested three indicators—spontaneous recovery, reinstatement, and renewal—to assess the erasure of fear memories. Their findings revealed that spontaneous recovery, reinstatement, and renewal were almost absent if immediate extinction was performed within 10 min to 1 h after fear memory acquisition. This finding suggests that performing immediate extinction within this time frame can lead to fear memory erasure (9). Such erasure can inhibit the consolidation of fear memories, preventing their transition into long-term memories. Notably, these results have been replicated in several other laboratories, demonstrating the efficacy of immediate extinction in fear memory erasure (11). However, the mechanisms underlying why immediate extinction can erase fear memories remain unclear. Additionally, the relatively short time window for immediate extinction has limited its application in clinical practice.

#### 2.1.2 Post-retrieval extinction

According to the theory of memory reconsolidation, memories become labile once again during the reconsolidation process after retrieval. Therefore, conducting extinction training within this reconsolidation timeframe may serve as another method to erase fear memories (12). Monfils et al. conducted pioneering experiments on post-retrieval extinction using the behavioral model of auditory fear conditioning in rats. They induced retrieval once 24 h after fear memory acquisition (i.e., presented CS once), followed by extinction training at various time points post-retrieval (i.e., 10 min, 1 h, and 6 h). Subsequent testing for spontaneous recovery, reinstatement, and renewal revealed the absence of these phenomena when extinction was performed within 10 min to 1 h after retrieval, indicating that extinction performed within this timeframe can erase fear memories (10). This effect may be a form of memory updating during reconsolidation and can only occur within the reconsolidation time window, typically within 6 h in rats (13, 14). Evidence supports this claim in animals. Using a similar behavioral model, Schiller et al. investigated the erasure of fear memory in healthy individuals. Participants were exposed to colored squares on a screen as the CS and received mild wrist shocks as the US. After fear conditioning, participants underwent training based on the post-retrieval extinction model. The results demonstrated memory erasure after extinction training conducted 10 min after retrieval (15). Subsequently, Yan-Xue Xue et al. successfully applied this model to erase memories of heroin addiction (16). Owing to its experimental success, this post-retrieval extinction model based on memory reconsolidation has promising clinical potential and attracted attention (17, 18); nonetheless, whether this behavioral method can be used to treat patients suffering from anxiety disorders and why it is effective for fear memory erasure remain unclear.

Traditional exposure therapy has considerable difficulties in effectively addressing fear memories, primarily due to the challenges posed by spontaneous recovery, reinstatement, and renewal phases, which often hinder complete memory eradication. Given that most clinical conditions like PTSD and anxiety disorders are long-standing, the narrow time window for immediate extinction limits its application in clinical settings. In contrast, post-retrieval extinction has demonstrated greater potential in modifying fear memories. However, while these approaches are promising, further extensive research is necessary to enhance their efficacy, particularly regarding the complete erasure of long-term fear memories.

## 3 Erasing short- and long-term memories

Although behavioral interventions have demonstrated the potential to erase fear memories, their effectiveness varies significantly between short-term and long-term memories. The following section explores strategies and challenges in addressing these two types of fear memory.

The most effective treatment method for erasing short-term memory is to apply the behavioral intervention method of post-retrieval extinction, which acts during the period of lability induced by memory retrieval. Johannes Gräff et al. conducted various fear extinction trainings in mice 1 and 30 days after fear memory acquisition to reduce their conditioned fear response. They discovered that extinction training conducted 1 day after fear memory acquisition could extinguish the fear memory, whereas the same training conducted 30 days later did not (19). While the post-retrieval extinction paradigm effectively erases short-term fear memories, it is less effective for long-term memories. This is because long-term fear memories form more stable synaptic connections and undergo stronger reconsolidation processes, making them harder to erase through behavioral interventions alone (20–22). Therefore, erasing long-term memories may require a combination of pharmacological and behavioral therapies to enhance neural plasticity and weaken these consolidated memories (23, 24). However, further investigation is required to elucidate why erasing long-term fear memory is more challenging than short-term fear memory.

## 4 Fear memory erasure and neuroplasticity

The challenge in erasing long-term fear memories underscores the importance of neuroplasticity, a key factor in memory formation and modification. Thus, we will explore how enhancing neuroplasticity may improve strategies for fear memory erasure.

Diminished neuroplasticity is a key factor contributing to the difficulty of erasing long-term fear memories. Neuroplasticity refers to the brain’s ability to undergo changes and reorganize itself to better adapt to new environments. After birth, an animal’s neural circuits undergo changes to adapt to the external environment. This process continues until the neural circuits and synapses are essentially fully established in adulthood, after which the brain structure no longer drastically changes, and plasticity is greatly reduced. However, brain structures critical to the formation of new memories remain plastic throughout an animal’s life. Consequently, changes in neuroplasticity are generally positive and adaptive, but they can sometimes be negative and maladaptive. For instance, reduced neurotransmitter levels, atrophy, and decreased synaptic connections are maladaptive manifestations of neuroplasticity (25). The formation of negative neuroplasticity is often associated with mental disorders such as stress, anxiety, schizophrenia, and PTSD (26).

Synaptic strengths can change with experience, a crucial aspect of the nervous system. In the 1970s, researchers discovered that the strength of connections between hippocampal neurons could be altered under high-frequency stimulation (27). Studies have demonstrated that fear conditioning, as a form of associative memory, can be deactivated and reactivated through long-term depression (LTD) and long-term potentiation (LTP), respectively (28). The amygdala is a critical brain area for fear regulation. Sadegh Nabavi et al. engineered through optogenetic techniques the inactivation and deactivation of memory through LTD and LTP, respectively, which led rats to associate a foot shock with optogenetic stimulation of auditory inputs targeting the amygdala. Notably, the optogenetic delivery of LTD conditioning to the auditory input inactivated the memory, and the optogenetic delivery of LTP conditioning to the auditory input reactivated the memory of the shock in the rats (28). Therefore, memory can be encoded by modifying synaptic strengths through cellular mechanisms such as LTP and LTD. In summary, the erasure of long-term fear memories is closely linked to neuroplasticity, with mechanisms like long-term potentiation and depression offering key insights for improving treatment efficacy.

## 5 Neural circuit mechanism of memory erasure

Building on the role of neuroplasticity in memory erasure, it is important to explore the neural circuit mechanisms that govern the extinction and potential erasure of fear memories. Understanding these circuits can offer valuable insights into improving therapeutic strategies for fear memory modification.

**Figure 1:**
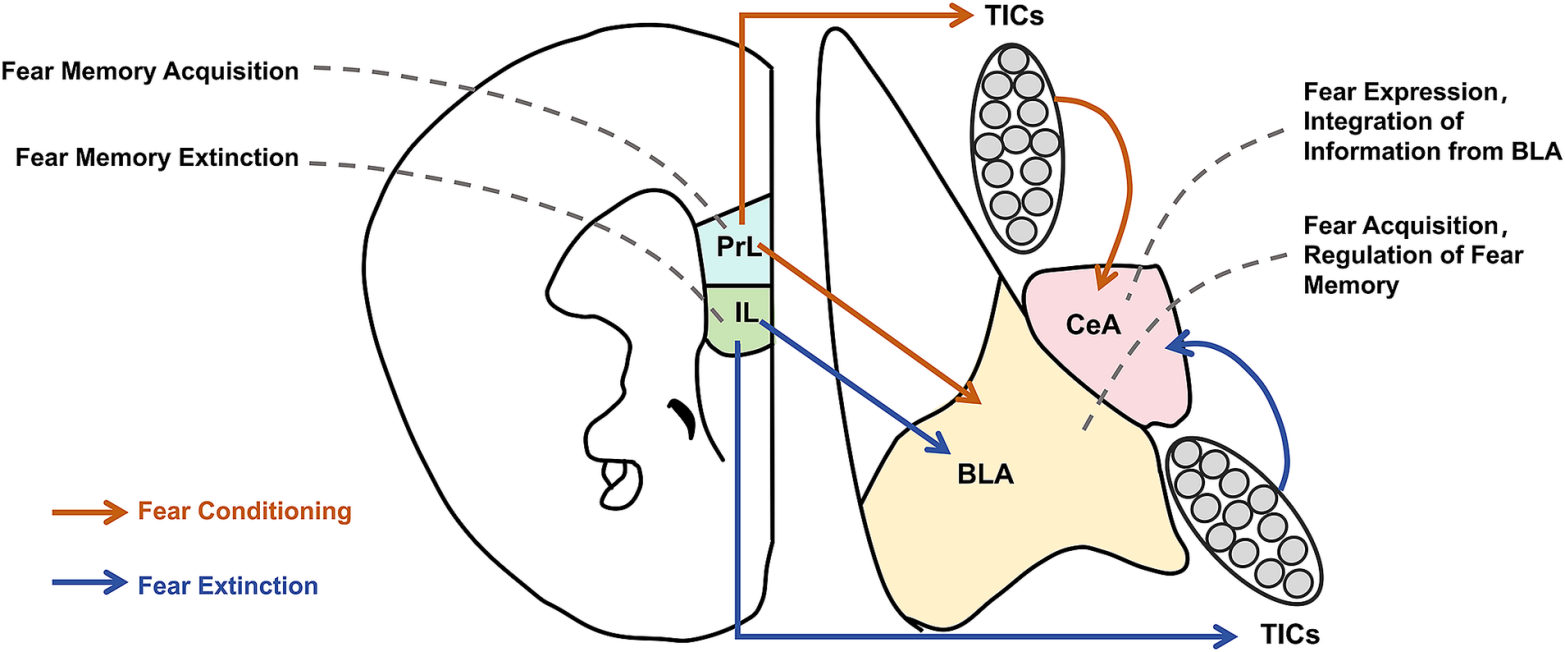
Neural circuit mechanism of fear memory erasure and acquisition. This figure illustrates the interactions of key neural circuits involved in the acquisition and erasure of fear memories. Brain regions such as the Prelimbic Cortex (PrL), Infralimbic Cortex (IL), Basolateral Amygdala (BLA), and Central Amygdala (CeA) interact through neurotransmitter modulation and signaling pathways, working together to regulate both the acquisition and elimination of fear memories.

Many studies have investigated the neural circuit mechanism of memory extinction, yielding notable results. In the context of auditory-cued fear memory, the core circuit of memory extinction may primarily involve the basolateral amygdala (BLA) and medial prefrontal cortex (mPFC). The BLA comprises the lateral nucleus of the amygdala and the basal nucleus of the amygdala (BA), and the mPFC mainly includes the prelimbic (PL) and infralimbic (IL) cortices of the prefrontal lobe (29–31). Individual suppression of these brain areas reveals their distinct roles in fear memory acquisition and extinction (Figure 1). Selectively inhibiting BLA, PL, and IL using the gamma-aminobutyric acid receptor agonist muscimol demonstrated that inhibiting BLA affects fear memory acquisition and extinction, inhibiting PL affects fear memory acquisition but not extinction, and inhibiting IL affects extinction but not fear memory acquisition (32). Additionally, strong bidirectional projections exist between BLA and mPFC, with mPFC projections modulating fear memories. Fear memory formation and extinction can increase the excitability of PL–BLA and IL–BLA projections, respectively. Further, inhibiting these projections can influence the retrieval of extinction memories (31, 33). Senn et al. combined circuit tracing and immediate-early gene (IEG) expression to reveal that BA neurons that project to PL and IL are fear and extinction cells, respectively (34). Klavir et al. performed the optogenetic induction of LTD, revealing that inducing LTD in BLA–PL and BLA–IL projections affects memory retention and promotes the extinction of fear memories (35). The dissociable functions of PL and IL in memory extinction may also be associated with IL projections to the intercalated cell masses (ITCs). Notably, memory extinction induces IEG expression in ITCs, which promotes memory extinction by inhibiting the lateral central amygdala (36–38). Moreover, IL–PL projections are crucial for fear memory extinction (39). Despite much research on the neural circuitry of memory extinction, the neural circuitry of fear memory erasure is poorly understood. However, as the behavioral method of fear memory erasure depends on memory extinction, similarities may exist between the neural circuitries of fear memory erasure and memory extinction. A study using the post-retrieval extinction model in humans found that the connection between the mPFC and BLA was attenuated in the post-retrieval extinction group compared with that of the extinction alone group, suggesting that changes in neural circuit plasticity between the mPFC and BLA represent a key mechanism in memory erasure mediated by post-retrieval extinction (40) (Figure 1).

Researchers have discovered that neuroplasticity within the mPFC is crucial for fear extinction by analyzing neural networks in the IL and PL regions (41). Borgomaneri et al. explored the role of the dorsolateral prefrontal cortex (dlPFC) in the reconsolidation of fear memories using state-dependent repetitive transcranial magnetic stimulation (rTMS). The researchers conducted six experiments with 84 healthy participants who underwent differential fear conditioning. rTMS was applied to the dlPFC 10 min after the fear memory was reactivated. The study found that applying rTMS to the dlPFC after memory reactivation significantly reduced physiological responses to fear memories and prevented the return of fear, highlighting the critical role of the dlPFC in the reconsolidation of fear memories (42).

Fear memories are not fixed; they can be “rewritten” or modified through the precise manipulation of neural circuits. Non-invasive brain stimulation techniques like TMS can modulate the activity of key brain regions involved in fear memory, such as the mPFC and BLA, facilitating the extinction or alteration of these memories. Additionally, optogenetics enables researchers to control neural network activity in experimental animals by selectively activating or inhibiting specific groups of neurons through targeted light stimulation. These studies highlight the potential of these techniques in understanding the mechanisms of fear memory and developing treatments for PTSD and provide a crucial theoretical foundation for future clinical applications (42–44).

In summary, research on the neural circuitry of fear memory extinction and erasure has revealed critical brain regions, such as the basolateral amygdala (BLA) and medial prefrontal cortex (mPFC), that play pivotal roles in memory processing. Manipulating these regions through techniques such as optogenetics and TMS has shown potential for altering or erasing fear memories. However, the exact mechanisms behind fear memory erasure are still not fully understood, and further exploration is needed to optimize these findings for clinical treatments, particularly for conditions like PTSD.

## 6 Molecular mechanisms of memory erasure

This chapter will explore the molecular mechanisms contributing to memory erasure, complementing the neural circuit mechanisms discussed earlier. Molecular processes such as epigenetic regulation, protein synthesis and degradation, and protein phosphorylation are fundamental in the processes of memory stability, extinction, and erasure. This section will examine the roles of these molecular factors in fear memory erasure and their potential implications for therapeutic applications.

### 6.1 Epigenetic regulation

Many recent studies have elucidated the role of epigenetic regulation in models of memory reconsolidation. For example, the histone acetyltransferase inhibitor garcinol inhibits the reconsolidation of cocaine-cue memories and suppresses conditioned reinforcement (45, 46). Shi et al. found that inhibition of methyltransferase activity in the BLA after memory reactivation inhibited conditioned and unconditioned memory reinstatement (47). Additionally, histone deacetylase inhibitors can enhance extinction learning, resulting in a similar outcome to post-retrieval extinction, where memories are not spontaneously reinstated after a prolonged period (48). Regarding the possible relationship between post-retrieval extinction and histone deacetylase, Johannes Gräff et al. found that the reason for post-retrieval extinction training for fear memory erasure may be related to increased nitrosylation of histone deacetylase 2 in the amygdala. Their study explains the relationship between fear memory erasure and histone deacetylase to some extent (19). Additionally, histone deacetylase inhibitors are well tolerated in anticancer therapies, with many of the side effects being reversible, suggesting promising applications for histone deacetylase inhibitors in clinical therapies (49). Firyal Ramzan et al. investigate the role of the histone variant H2A.Z in the androgen receptor (AR)-mediated regulation of fear memory. They found that H2A.Z is crucial for AR’s ability to reduce fear memory (50). Researchers investigated the role of the IQGAP1/ERK signaling pathway in the formation of fear memory. They found that IQGAP1 interacts with GluN2A-containing NMDA receptors and the ERK1/2 cascade, regulating histone H3S10 phosphorylation and H3K14 acetylation, thereby influencing the formation of fear memory. Additionally, inhibiting HDAC2 can restore fear memory, highlighting the critical regulatory role of HDAC2 in this process. This indicates that the IQGAP1/ERK signaling pathway can regulate fear memory through HDAC2-induced post-translational modifications of histones (51).

### 6.2 Protein synthesis and degradation

**Figure 2:**
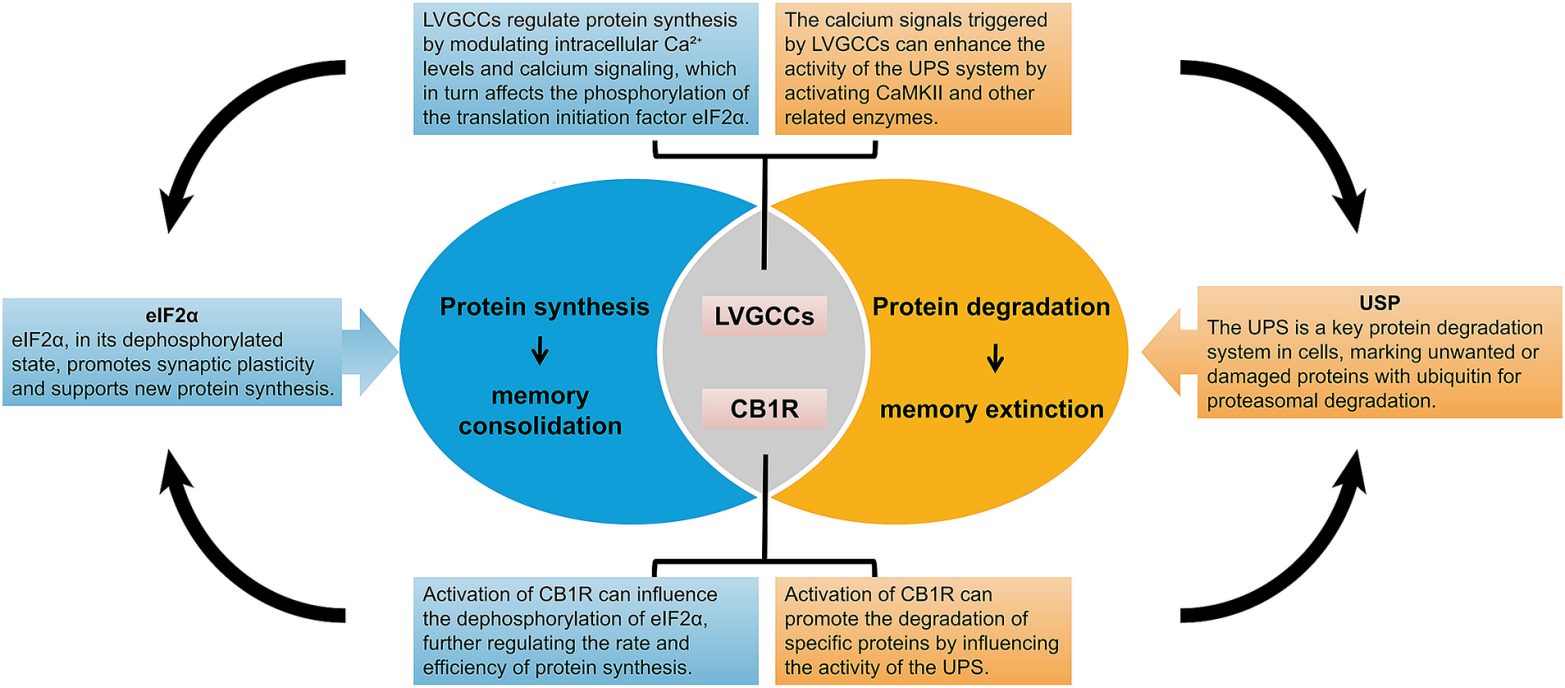
Mechanisms of protein synthesis and degradation in memory consolidation and extinction. This figure illustrates the molecular mechanisms underlying protein synthesis and degradation in memory consolidation and extinction. It highlights the roles of key components such as L-type voltage-gated calcium channels (LVGCCs), CB1 receptors (CB1R), Ubiquitin-Specific Protease (USP), and eIF2α in regulating protein synthesis. The figure also emphasizes how these molecular processes contribute to the stabilization and extinction of memories.

As memory formation is often accompanied by the synthesis of new proteins, inhibition of protein synthesis has been considered an important mechanism for memory erasure, and many studies have been conducted in this regard. For example, the use of protein synthesis inhibitors such as anisomycin, cycloheximide, and rapamycin can inhibit memory reconsolidation (52). Protein synthesis has been considered essential for memory consolidation and reconsolidation, and protein degradation is believed to be associated with memory destabilization (53). Lee et al. found that the level of polyubiquitination of proteins in the CA1 region of the hippocampus was significantly increased at specific times after reinstatement of contextual fear memories and that ubiquitin-proteasome system (UPS)-dependent protein degradation plays an important role in destabilization after memory reinstatement (54). Suzuki et al. found that inhibiting protein synthesis in a contextual fear conditioning paradigm destabilized memory restabilization after reactivation and that the blockade of L-type voltage-gated calcium channels (LVGCCs) or cannabinoid receptor 1 receptors (CB1R) prevented the amnestic effects caused by inhibition of protein synthesis at the memory destabilization stage (55). LVGCCs activated via synaptic activity mediate a large influx of Ca^2+^ ions that activates CAMKII, which phosphorylates and enhances the hydrolytic activity of the proteasome and regulates proteasome repositioning, affecting local UPS activation (56). Injection of the proteasome inhibitor lactacystin into the nucleus accumbens core protects against memory impairment induced by the anisomycin injection. This treatment also maintained the synaptosomal expression of the AMPA receptor subunit GluR2 (57). Collectively, these studies suggest that fear memory erasure may be related to proteasome-mediated protein degradation (Figure 2).

Eva Mª Pérez-Villegas and her colleagues (58, 59), through studies using HERC1-mutant mice (tbl mice), discovered that HERC1 mutations impair synaptic plasticity and structural stability in the lateral amygdala. This includes the loss of LTP, an increased proportion of immature dendritic spines, and reduced glutamatergic transmission, ultimately leading to deficits in associative learning. These findings highlight that HERC1 is in learning and memory by regulating protein homeostasis. The study found that during the consolidation and reconsolidation of fear memory, proteasome activity and linkage-specific polyubiquitination patterns within the amygdala exhibit significant and distinct changes across different subcellular compartments. For example, in certain subcellular locations, proteasome activity significantly increases, while in others, it decreases, and the types of polyubiquitination linkages also vary. This indicates that the UPS not only regulates the overall stability and function of synaptic proteins but also finely tunes the consolidation and reconsolidation processes through specific regulation within different subcellular compartments (60). The study found that the protein synthesis initiation factor eIF2α significantly influences memory consolidation through cell-type-specific translational control in excitatory neurons and somatostatin (SST) expressing inhibitory neurons. Dephosphorylation of eIF2α enhances LTP and long-term memory (LTM) without affecting short-term memory (STM). This suggests that eIF2α acts as a molecular switch, playing a critical role in memory consolidation by modulating translation in a cell-type-specific manner (61, 62). These studies elucidate the essential roles of protein synthesis and degradation in both memory formation and extinction, providing new theoretical foundations and experimental evidence for the potential erasure of fear memories (Figure 2).

### 6.3 Protein phosphorylation

Various protein kinases regulate memory reconsolidation. Intracellular signaling pathways that regulate neuronal plasticity, such as those of MAPK/ERK, PI3K, and second messengers, are involved in the regulation of memory reconsolidation and extinction. Fear memory reconsolidation is dependent on ERK activity in the BLA. Additionally, inhibition of CaMKIIα in the BLA inhibits reconsolidation and enhances the extinction of fear memories (63–65). Yang and Lu found that weak extinction training induced small increases in P-MAPK and P-AKT in the BLA, and concomitant administration of the NMDA receptor agonist D-cycloserine (DCS) significantly increased the level of P-MAPK and P-AKT signaling, suggesting that DCS may promote memory extinction through the P-MAPK and P-AKT signaling pathways (66). Consistent with these results, intra-BLA infusion of the MAPK inhibitors U0126 or PD98059 or the PI3K inhibitor wortmannin blocks the facilitation of memory extinction via DCS. Targeted infusion of the transcriptional inhibitor actinomycin D in the BLA also blocked the DCS-induced facilitation of extinction (67). These studies demonstrate the role of protein phosphorylation and signal transduction in memory reconsolidation. Notably, memories cannot form without protein phosphorylation being affected (68); therefore, the relationship between memory erasure and protein phosphorylation and signal transduction and whether memory erasure can be achieved by targeting these connections require further exploration. Gabriele Musumeci and his colleagues (69) discovered that the TrkB receptor regulates fear learning and amygdalar synaptic plasticity through its two critical phosphorylation sites, Y515 and Y816. This indicates that different signaling pathways of the TrkB receptor play specific roles in the formation and consolidation of fear memory.

In summary, the molecular mechanisms involved in memory erasure, including epigenetic regulation, protein synthesis and degradation, and protein phosphorylation, play pivotal roles in memory stability, extinction, and reconsolidation. Epigenetic modifications, such as histone acetylation and methylation, influence the consolidation and retrieval of fear memories, providing potential therapeutic targets for memory modification. Protein synthesis and degradation, particularly through the ubiquitin-proteasome system, contribute to memory destabilization, and phosphorylation of key proteins regulates neural plasticity and memory extinction. These molecular processes underscore the complex interplay that governs memory erasure and highlight promising avenues for therapeutic interventions aimed at modifying or erasing unwanted memories (Table 1).

**Table 1:** Overview of molecular mechanisms in memory erasure.

Acting factors	Routes of action/effects	References
Garcinol	Inhibits reconsolidation of cocaine-cue memories and suppresses conditioned reinforcement.	(45, 46)
Methyltransferase inhibition	Blocks conditioned and unconditioned memory reinstatement in the BLA after memory reactivation.	(47)
Histone variant H2A.Z	Critical for androgen receptor-mediated reduction of fear memory.	(50)
IQGAP1/ERK signaling pathway	Modifies histones (H3S10 phosphorylation, H3K14 acetylation) to regulate fear memory formation.	(51)
Protein synthesis inhibitors	Block memory reconsolidation, showing the importance of protein synthesis for memory processes.	(52, 53)
Ubiquitin-proteasome system (UPS)	Regulates memory destabilization and synaptic protein stability via polyubiquitination during fear memory.	(54, 60)
LVGCCs and CAMKII	Activate proteasome activity through Ca^2+^ signaling, influencing local UPS regulation in memory processes.	(56, 60)
Proteasome inhibitor (lactacystin)	Prevents anisomycin-induced memory impairment and maintains AMPA receptor GluR2 expression.	(57)
HERC1 mutations	Impair synaptic plasticity, reduce glutamatergic transmission, and disrupt fear memory consolidation.	(58–60)
eIF2α (dephosphorylation)	Enhances LTP and long-term memory through cell-type-specific translational control.	(61, 62)
CaMKIIα inhibition in the BLA	Blocks reconsolidation and enhances extinction of fear memories.	(63–65)
D-cycloserine (DCS)	Promotes extinction via P-MAPK and P-AKT signaling in the BLA.	(66)
TrkB receptor phosphorylation	Y515 and Y816 phosphorylation sites regulate fear memory formation and synaptic plasticity in the amygdala.	(69)

## 7 Engram cells in fear memory erasure

To better understand the mechanisms behind memory erasure, it is crucial to examine the role of engram cells, the neural substrates that store and retrieve memories. These specialized cells not only help maintain memories through strengthened synaptic connections but also play a key role in the reactivation and erasure of memories. In this section, we will explore the concept of engram cells in the context of fear memory erasure, examining how the manipulation of these cells can lead to the modulation or erasure of established memories.

The term “engram” was introduced by the German biologist Richard Semon in 1904 to describe the neural substrate involved in memory storage and retrieval. Semon reported that experienced events activate a group of neurons, leading to chemical or physical changes that constitute an engram. Notably, the specialized cells involved in this were referred to as engram cells, and their subsequent reactivation by partial cues induces memory retrieval. However, after Karl Lashley failed to identify such engrams in the brains of rats, further attempts to locate engrams in the brains were largely abandoned (70). Nevertheless, the impetus to conduct engram-related research was revived by Karl Lashley’s students, Brown RE and Donald O. Hebb, who proposed cell assembly theory. This theory suggests that intrinsically linked cells activated by the same event form cell assemblies with strengthened synaptic connections, often summarized as “cells that fire together, wire together” (71). These engrams, composed of specific neuron assemblies, maintain memory through strengthened synaptic connections. Researchers have introduced the concept of “silent” engrams, which can be reactivated through artificial stimulation even when natural cues fail. Additionally, engrams are complex, with a single memory potentially supported by neuron assemblies across multiple brain regions (72). Recent advances in experimental techniques, particularly in transgenic mice, have facilitated engram cell research using methods such as immunofluorescence labeling and optogenetics, yielding notable results. These studies have focused on observing engrams, studying engram loss of and gain of function, and exploring engrams’ roles in memory processes (73, 74).

Observing engrams involves using IEGs (e.g., c-Fos, Arc, or Zif268) to visualize neuronal activation (72, 75). Notably, Mayford et al. were the first to develop the TetTag transgenic mouse, a bi-transgenic mouse expressing tTA driven by the Fos promoter, and tauLacZ driven by the tTA*–TetO. This mouse can be controlled by doxycycline (Dox), wherein tauLacZ is not expressed in the presence of Dox, but in its absence, tauLacZ expression is driven by Fos. Therefore, Dox can be used to label neurons activated within a specific time window. Specific labeling of activated neurons is achievable using these transgenic mice (76).

Investigations into engram loss of function were pioneered by Jin-Hee Han et al., whose previous research demonstrated that neurons with high CREB expression were more likely to be allocated to engrams. Hence, they first utilized CREB overexpression to allocate engrams to neurons overexpressing CREB in the lateral amygdala (LA). Subsequently, after auditory fear conditioning, they used diphtheria toxin combined with the Cre–Loxp system to selectively induce the apoptosis of CREB-overexpressing neurons in LA, which resulted in impaired auditory fear memory (77). Subsequently, other researchers utilized Daun02 combined with c-fos-lacZ transgenic rats in a cocaine-induced addiction memory model to selectively inactivate engram cells for addiction memory in the nucleus accumbens. Notably, the inactivation of engram cells could impair addiction memory (78). Additionally, Anthony F. Lacagnina et al. discovered that suppressing the engram cells of context-dependent fear memory in the dentate gyrus using optogenetics also impaired the expression of fear memory (79). These experiments collectively demonstrate the essential role of engram cells in memory expression.

Research on engram gain of function by Xu Liu et al. employed optogenetics to activate the engram cells of context-dependent fear memory in a non-training context, which elicited freezing in mice. The study highlighted that even without training within this context, artificial activation of the engram cells sufficed to induce the retrieval of context-dependent fear memory (80). Subsequently, a similar experiment demonstrated that optogenetic or chemogenetic reactivation of labeled or allocated engram cells in various brain regions could evoke memory expression without additional sensory cues (81), confirming the sufficiency and necessity of engrams for memory expression.

To explore how engrams change in memory erasure, Anthony F Lacagnina et al., by labeling contextual fear engram cells in the DG and optogenetic experiments, demonstrated that spontaneous recovery after memory extinction is due to the reactivation of fear memory cells at the time of memory acquisition, and extinction engrams are encoded in different cellular ensembles than memory acquisition engrams (79). However, they did not focus on the relationship between extinction and acquisition engrams. Another study suggested that activation of the original fear engram cells in the hippocampus facilitates remote contextual fear memory erasure and that activation of engram cells in the hippocampus before extinction prevented the spontaneous recovery of the memory (82). Moreover, another study reported increased reactivation of engram cells in the prelimbic cortex and BLA during memory updating. Additionally, memory updating initiated by conditioned and unconditioned stimuli depends on the reactivation of engram cells in the prelimbic cortex and BLA, respectively. Finally, memory updating causes increased overlapping between fear and extinction cells, and the original fear engram encoding is also altered (83). These experiments suggest that the nature of memory erasure may be an alteration of the original engram cells. In contrast, memory extinction alone might not alter the original engram cells, and the original engram cells can be reactivated during retrieval, inducing a fear memory.

In summary, engram cells are crucial for the storage, retrieval, and modulation of fear memories. Through advanced techniques such as optogenetics and transgenic mice, researchers have shown that manipulating these cells can significantly influence memory expression and erasure. The activation or inactivation of engram cells, whether in the amygdala, hippocampus, or other brain regions, highlights their importance in both the formation and extinction of fear memories. These findings provide valuable insights into the potential for targeting engram cells in therapeutic approaches aimed at memory modification or erasure.

## 8 Perineuronal net and fear memory erasure

To further understand the mechanisms underlying memory erasure, it is essential to consider not only the molecular processes but also the structural components that influence memory storage and modification. One such component, the perineuronal net (PNN), is crucial in regulating the stability and plasticity of neural circuits involved in memory. In this section, we will examine the impact of PNNs on fear memory erasure, exploring how their degradation or manipulation may contribute to the modification of established memories.

Studies on sensory systems have highlighted the importance of inhibitory neural circuits in brain plasticity. In animal experiments, monocular deprivation during early visual cortical development enhanced visual function in the non-deprived eye compared with that in the deprived eye. However, this effect diminished when monocular deprivation occurred after the critical period of neuroplasticity (84). PNNs may be a key factor affecting the critical period of neuroplasticity. These specialized structures, consisting mainly of chondroitin sulfate proteoglycans (CSPGs) within the extracellular matrix, are primarily found around interneurons, with some presence around pyramidal neurons (85). The development of PNNs in the visual cortex aligns closely with the critical period of neuroplasticity, suggesting their significance in this process (86). PNNs are also present in the BLA (87). Gogolla et al. demonstrated that degrading PNNs using chondroitinase ABC erased fear memories through extinction training, indicating that PNNs can protect acquired fear memories from erasure (88). Studies have shown that memory formation coincides with the formation of PNNs around memory cells, and the degradation of these PNNs affects memory consolidation (89). As PNNs predominantly surround inhibitory neurons, they probably affect neuroplasticity by altering local inhibitory circuits, influencing the acquisition and erasure of fear memories.

Liu et al. investigated the role of perineuronal nets (PNNs) in the CA1 region of the hippocampus in retaining long-term contextual fear memory. Their research found that removing PNNs using the enzyme ChABC led to reduced firing rates of parvalbumin neurons, decreased inhibitory synaptic transmission, and impaired retention of long-term contextual fear memory, while short-term memory remained unaffected. These findings suggest that PNNs are crucial for retaining fear memory by regulating presynaptic GABA release (90). In a related study, Angelina Lesnikova et al. explored the role of the PNN receptor protein tyrosine phosphatase sigma (PTPσ) in memory retention. Their study revealed that PTPσ regulates memory retention by dephosphorylating the TRKB receptor, which, in turn, limits neural plasticity. Mice with a deletion of the PTPσ gene exhibited improved short-term memory but impaired long-term memory retention, indicating that PTPσ has distinct regulatory functions at different stages of memory. The interaction between PNNs and PTPσ presents a potential target for treating memory-related disorders (91). These studies emphasize the critical role of PNNs in retaining long-term fear memory.

In summary, PNNs are essential in the maintenance and modulation of fear memories. By surrounding inhibitory neurons, they influence the plasticity of neural circuits, thereby regulating the formation and retention of memories. PNNs protect acquired fear memories from premature erasure and help stabilize long-term memory retention through changes in local inhibitory transmission. Disruption or degradation of PNNs can lead to the loss of fear memories, highlighting their essential role in both memory consolidation and erasure. Therefore, PNNs represent a promising target for therapeutic interventions aimed at memory modulation and related disorders.

## 9 Clinical strategies and technological advances in fear memory erasure

The clinical application of fear memory erasure has made significant advances, with various innovative approaches showing potential for treating disorders such as PTSD. Building upon advancements in molecular and neural mechanisms discussed earlier, these clinical strategies focus on translating foundational research into effective therapeutic interventions. Among these, TMS has emerged as a promising non-invasive neuromodulation technique. By using electromagnetic pulses to precisely regulate neuronal activity in cortical regions, TMS has proven to be an effective, safe, and well-tolerated treatment for psychiatric disorders, including PTSD (92, 93). Its increasing adoption in clinical practice highlights its therapeutic value.

In parallel, advanced technologies such as functional magnetic resonance imaging (fMRI) combined with optogenetics are paving the way for more precise interventions. These tools allow researchers to track the dynamic changes in fear memory networks in real-time, offering detailed insights into the formation, modification, and potential erasure of fear engrams (94, 95). Particularly, optogenetics has emerged as a powerful approach for manipulating neural circuits with high precision. By integrating genetic and optical methods, optogenetic interventions can selectively weaken trauma-related fear memories and restore normal neural functions, as demonstrated in animal models (96).

Despite the effect of the neuromodulation technique is powerful, the durability of long-term effects were not very well. Structural interventions, such as degrading PNNs to enhance neural plasticity, also present promising avenues for fear memory erasure (97, 98). However, these approaches come with risks, such as destabilizing critical neural circuits, underscoring the need for careful optimization. As these techniques continue to evolve, they hold great promise for transforming the treatment of PTSD and other fear-based disorders by providing more targeted, effective, and individualized therapies.

## 10 Discussion

Based on the results of the previous research, the following conclusions can be drawn: Post-retrieval extinction currently stands as the predominant paradigm for memory erasure. This paradigm demonstrates efficacy in erasing short-term memory but proves inadequate for long-term memory. As many patients with clinical PTSD have difficulty with long-term fear memories, the primary challenge is whether this paradigm is effective for anxiety disorders, PTSD, and related conditions in clinical practice. Moreover, research on the mechanism of memory erasure is relatively scarce, with most studies focusing on the mechanisms of memory extinction, consolidation, and reconsolidation, and a plausible explanation for the essential difference between memory erasure and memory extinction remains lacking.

In auditory-cued fear memory, the core circuit of memory extinction is thought to primarily involve the BLA and mPFC. Additionally, strong bidirectional projections exist between BLA and mPFC, which are responsible for modulating fear memories. A study found that changes in neural circuit plasticity between the mPFC and the BLA represent a key mechanism in memory erasure mediated by post-retrieval extinction (40). However, changes in these circuits during memory erasure and whether other brain regions or circuits are involved remain unexplored. The advances in genetic modification tools and transgenic mice, as well as experimental methods for labeling neuronal activity, such as *in vivo* Ca imaging, have contributed to a growing body of research on cellular encoding, which may offer a new perspective to explain the essence of memory erasure. Studies have shown that the encoding of the original fear engram is altered during the memory updating process. Hence, whether cellular encoding affects the erasure of long-term fear memory will probably be resolved in further investigations.

As fear memory erasure is based primarily on the theory of memory reconsolidation, most recent studies on the molecular mechanisms of memory erasure have also focused on the molecular and biochemical changes that occur during memory reconsolidation. Many studies have elucidated some of the alterations in the processes of memory reconsolidation and fear memory erasure from multiple perspectives and mechanisms, including epigenetic regulation, protein synthesis and degradation, and protein phosphorylation. Despite the extensive research on neuroplasticity, with particularly significant progress in molecular mechanisms, further comprehensive investigation is necessary to explore the molecular mechanisms of various types of synaptic plasticity. Additionally, studies have shown that PNNs may influence the erasure of fear memories, but the precise mechanisms remain unclear. Hence, intensifying research on fear memory and its molecular mechanism is imperative to provide novel insights into the treatment of PTSD, anxiety, and other mental disorders.

The erasure of pathological fear memories remains a challenging yet essential goal for addressing trauma-related disorders such as PTSD and severe anxiety disorders. Behavioral interventions, including exposure therapy, immediate extinction, and post-retrieval extinction, have shown varying degrees of efficacy, but their long-term durability and applicability in real-world clinical settings remain limited. The recurrence of fear memories through phenomena such as spontaneous recovery, reinstatement, and renewal highlights the difficulty of achieving complete and permanent memory erasure. Emerging molecular and structural approaches, such as epigenetic regulation, protein synthesis and degradation targeting, and perineuronal net (PNN) modulation, offer promising avenues but require further validation to ensure their long-term safety and effectiveness. Exploring combination therapies also holds significant potential, particularly by leveraging the synergistic effects of pharmacological agents and behavioral interventions, such as pairing post-retrieval extinction with neural plasticity-enhancing drugs like D-cycloserine. While substantial progress has been made in understanding the mechanisms of fear memory erasure, translating these insights into safe, effective, and durable clinical applications will necessitate interdisciplinary collaboration. Future research, driven by technological innovation and patient-centered approaches, could transform the treatment of trauma-related disorders.
